# Course of Lectures on the Physiology and Pathology of the Central Nervous System

**Published:** 1861-09

**Authors:** 


ART. IV.—*Course of Lectures on the Physiology and Pathology of the Central Nervous System.* Delivered at the Royal College of Surgeons of England, in May, 1858, by C. E. BROWN-SÉQUARD, M.D., F.R.S, etc., etc. pp. 276. Philadelphia: J. B. Lippincott and Co., 1860.

THE immense progress made by the physiology of the last fifty years is in no one respect better exhibited than in its conquests over many of the difficulties with which nature has environed the study of the laws of the nervous system. In this embarrassing pursuit, where chemistry has lent no aid, where the physics of the outer world has given no help, progress has been at all times slow, and the bones of dead and hasty theories lie thick upon the way.

Not the less surely has the general result been a gradual advance in the assured knowledge of important facts. Thus, while our conceptions as to the real nature of nerve force have advanced but little in the last thirty years, our more legitimate and hopeful efforts to study the laws which govern its manifestations in various portions of the nervous system have been as satisfactory as the nature of the subject should allow us to look for. Whenever, indeed, nerve trunks and nerve centres were sufficiently isolated to admit of careful and distinct examination without risk to neighboring organs, the methods of modern research have been amply rewarded; while, on the other hand, the nerve centres which lie closely grouped together have given but obscure answers to the questioning of the vivisector and pathologist. While thus we have had, comparatively speaking, but little difficulty in determining the functions of nerve trunks, and even of certain nerve centres, the physiology of most of the groups of gray matter in the encephalon has hitherto baffled the biologist in his efforts at minute functional definition, and even the group of spinal centres, with its connective and covering strands of white neurine, has never yet been satisfactorily explored, although its position and relations seem to offer facilities which disappear when we attempt to investigate the complicated mechanism of the upper ganglia.

Since the time of Sir Charles Bell, no physiologist has done more distinct service to the cause of spinal neurology than the author whose name stands at the head of this article, while in numerous other branches of physiology he has also rendered material aid.

In offering our readers an analysis of the volume before us, it is not our intention to criticise its results. The author is one who has given to his work many years of labor. He is acknowledged to be among the most cautious and careful of experimentalists, and, whether in the conduct of his researches, in the care with which he examines the results of vivisections, or in the industry with which he collects pathological and other illustrations of his views, he is unsurpassed by any of the biologists of the day. In one particular also the present volume, and indeed all of his writings, are deserving of praise which can scarcely be accorded on like grounds to some of his contemporaries. Every statement and every experiment not distinctly his own is fairly referred to its author, so that we learn precisely to what extent he presumes his own experiments in any particular case to have advanced the subject beyond the current knowledge of the day. The most remarkable contrast to this honest style of treating subjects is to be met with in the various courses of lectures which M. Bernard has published within the last few years. In all of these there is of course much that is purely original, but so much that is not so is stated as though it were, that a tyro might presume the author to have discovered and illustrated every known law of physiology. Now it is not credible that such an impression or anything like such an impression is what M. Bernard desires to produce; but this only is sure, that no one, however learned in physiology, can read his lectures without continually feeling this defect. Nor is this deficiency confined to the works of biologists so eminent as M. Bernard; it has crept in worse form into the pages of one of our popular writers whose volumes convey to the unprofessional reader an idea of originality in biological research which is altogether undeserved.*

We have said, before thus digressing, that we did not intend to criticise the statements of M. Brown-Séquard. In examining the views of a physician writing on any of our ordinary maladies, every one has the warrant of some individual experience, and thus far is on even ground with him whom he criticises; but where the author treads new fields of research, and guides his steps by means which are unfamiliar, those only who have followed his steps with care are fitted to scrutinize the conduct of his journey.

All that we can do now is to report his opinions and wait until the experimental physiologists of our time have practically examined the foundations on which these views repose.

The present volume contains the result of twenty years of eager labor, and is divided into twelve lectures and an appendix.

Lecture I. defends Sir C. Bell's theory of the existence of two distinct sets of nervous conductors—the sensitive and the motor—and discusses the nature of the so-called recurrent sensibility of the anterior nerve roots, which the author thinks is merely the pain caused by the spasms of such muscles as are thrown into violent action when the anterior or motor roots are irritated. Lecture II. shows that the transmission of the sensitive impressions in the spinal cord takes place chiefly in its central part or in the gray matter. Two important facts are relied upon to support this proposition, namely, that a transverse section of the posterior white columns of the cord causes hyperæsthesia and not loss of sensibility, and that section of the whole cord, except the posterior column, is followed by complete anæsthesia. Lecture III. defends the author's well-known discovery of the decussation of the sensitive fibres in the whole length of the cord.

Lecture IV. still further discusses various questions relating to the transmission of sensitive impressions, and of the orders of the will to muscles, through the spinal cord and medulla oblongata. In this section numerous experiments are related to show that the various sensitive impressions of touch, pain, temperature, muscular contraction, etc. are transmitted by distinct conductors, which all decussate laterally in the cord, and which

* Lewes's Physiology of Common Life.

are so arranged that a large part of the cord, both white and gray, is engaged in carrying sensitive impressions; the conductors from any one part being so distributed in the spine that only very deep injuries can wholly destroy the sensation of a part. According to our author it is much more difficult to determine what are the parts of the cord employed in voluntary action, than to find out what are those through which sensitive impressions are forwarded. He finally arrives at the conclusion that in the dorsal region the various parts of the spinal cord, except the posterior columns, seem to be employed in the conveyance of the orders of the will to muscles; and that in the upper part of the cervical region of the cord, near the medulla oblongata, most of the conductors of the orders of the will to muscles are in the lateral columns and in the gray matter between these and the anterior columns. The volitional fibres decussate at the anterior pyramids, and there only.

In Lectures V., VI., VII., and VIII., M. Brown-Séquard quotes and analyzes a large number of cases bearing upon his views of spinal physiology. At p. 136 he thus sums up the chief symptoms which indicate disease of the spinal cord :—

"1st. *Deep alterations of the posterior columns in all their length.*—Increased sensibility in the trunk and limbs for impressions of touch, or to pricking, pinching, and galvanic excitations, and for changes of temperature, (cold and heat;) loss, or a very great diminution of reflex movements; all kinds of voluntary movements possible, and more or less easily executed when the patient is in bed; walking and standing very difficult.

"2d. *Deep alterations of the posterior columns in the extent of the cervico-bronchial swelling.*—Increased sensibility in the four limbs and in the trunk, for all kinds of impressions; diminution of reflex actions in the upper limbs, and increased reflex actions in the lower limbs; some difficulty in the direction of the movements of the upper limbs, without the help of the sight; standing and walking possible without any great difficulty.

"3d. *Deep alterations of the posterior columns in the extent of the dorso-lumbar swelling.*—Increased sensibility in the lower limbs and normal sensibility in the upper ones; diminution or loss of reflex actions in the lower limbs; movements of lower limbs possible, and even easy, when the patient is in bed; but walking and standing very difficult.

"4th. *Deep alteration of a very limited portion of the posterior columns.*—Increased sensibility and increased reflex action in all parts receiving their nerves from the spinal cord below the alteration; voluntary movements possible, and even easy, everywhere; the place of the alteration may be detected by diminution of reflex actions in the zone round the body receiving nerves from the level of the part altered in the posterior columns.

"5th. *Alteration of the posterior columns and posterior roots of the spinal nerves.*—Instead of hyperæsthesia, as in the preceding cases, diminution, or loss of all kinds of sensibility, in places receiving the spinal nerves, which are the continuation of the altered roots; voluntary movements still possible in bed, and while the patient looks at his limbs, but walking and standing almost impossible; reflex actions *completely* lost in all the anæsthetic parts; if the alterations are in the upper part of the spinal cord, the other parts being healthy, their voluntary movements in the lower limbs, and even walking or standing, are possible, and may be easy, and these limbs have an increased sensibility and increased reflex actions.

"6th. *Alteration of the posterior columns and of the gray matter in all their length.*—There is no difference between this case and the preceding, except that here there is a real paralysis of voluntary movements, which is complete if the alteration extends to the anterior gray cornua; greater frequency of formication and of other sensations referred to the periphery.

"7th. *Alteration of the posterior columns and gray matter in any limited part of the spinal cord.*—Very nearly complete loss of sensibility; degrees of paralysis of voluntary movements varying with the place occupied by the alteration in the length of the spinal cord; reflex actions increased in parts receiving their nerves from the portions of the cord below the seat of the alteration.

"8th. *Alteration limited to the gray matter.*—The same symptoms as in the preceding cases, except that at first there is a greater degree of anæsthesia than of paralysis, if the alteration begins in the very centre of the cord. Formication and other sensations referred to the periphery in cases of inflammation.

"9th. *Alteration of the anterior columns in the upper part of the cervical region.*—No paralysis; anæsthesia; very slight hyperæsthesia; various sensations (particular pain) referred to several parts of the body.

"10th. *Alteration of the lateral columns in the upper part of the cervical region.*—Paralysis of voluntary movements in the four limbs and the trunk; increased sensibility and greatly increased reflex actions in the paralyzed parts.

"11th. *Alteration of the anterior columns in any part of their length, except the neighborhood of the medulla oblongata.*—More or less complete paralysis of voluntary movements in all the parts receiving their nerves from or below the parts of the cord where the alteration exists; slight hyperæsthesia; reflex actions very much diminished in the parts which receive their nerves from the altered portion of the cord, and increased below these parts.

"12th. *Alteration of the lateral columns in any part of their length, except the neighborhood of the medulla oblongata.*—Incomplete paralysis of movements; hyperæsthesia; diminution of reflex actions less than in the preceding case.

"13th. *Alteration of the anterior half of the spinal cord, including the anterior columns, a good part of the gray matter, and a part of the lateral columns.*—Voluntary movements completely paralyzed; sensibility very much diminished. For reflex actions, as in 11th.

"14th. *Alteration of the various parts of the spinal cord, except the posterior columns.*—Loss of voluntary movements and of all kinds of sensibility; reflex actions increased or diminished in certain parts of the body, according to the place of the alteration in the length of the spinal cord."

Lecture IX. is devoted to the consideration of the sympathetic nervous system. The author establishes the facts that section of the cervical branch of this nerve causes dilatation of the blood-vessels of the corresponding side of the head, afflux of blood, and increase of vital properties, while galvanization of the cut extremity (the upper end) produces the reverse effects. M. Brown-Séquard states that the cervical and the cerebral sympathetic originates from the spinal cord as low as the tenth dorsal vertebra, Waller and Budge having placed the organic higher up, between the sixth cervical and fourth dorsal. He is also of opinion that the vaso-motor nerves of the lower part of the body come chiefly from the cerebro-spinal centres, as we think he has shown from the effect of sections of the dorsal and lumbar cord. The first experiment which links these physiological results to pathology and enables us to assert that reflex impressions may cause dilatation and contraction of vessels through their vaso-motor nerves, thus imitating the influence of section and galvanization of the sympathetic, is the well-known one of Dr. Tholozan, who showed that when the hand is placed in cold water, the blood-vessels of the other hand contract. The author next shows that section of a lateral half of the spine causes paralysis of the vessels of the limb on that side, and contraction of those on the other. Spasm of the capillaries is therefore to be regarded as a possible pathological incident, and starting thus, our author proceeds to apply this knowledge to explain certain cases of coldness in the limbs, some forms of gangrene, and other symptoms noticed in cholera and ague.

Lecture X. is perhaps one of the most valuable in the volume to the physician, although it contains least that is really novel. In it the author gives us a most complete resumé of cases of normal and abnormal reflex effects, causing healthy or unhealthy excesses or aberrations of secretions. He also considers very fully the causation of various forms of malady by reflex impressions, and gives at length his views as to the utilization of normal reflex agencies in the management of disease.

In Lecture XI. the author discusses his own well-known ideas as to the spinal origin of epilepsy, and the experiments by whose aid he has shown that, after certain injuries to the cord, an artificial epilepsy may be induced.

The aura-epileptica receives unusual attention. It appears that in many cases an imperfect aura really exists and may be demonstrated. Usually this is located where the first convulsive movements of the fit occur, or its site may be found by examining galvanically the surface of the body. Sometimes the application of ligatures to one limb at a time may determine where it begins, through the consequent cessation of the commencing symptoms of an attack.

Finally, he collates with his own results the important researches of Kussmaul and Tenner, and arrives at the following conclusions as to the causes and effects in epileptic seizure:—

| CAUSES. | EFFECTS. |
|---|---|
| 1. Excitation of certain parts of the excito-motory side of the nervous centre. | 1. Contraction of blood-vessels of the brain proper and of the face; spasm of some muscles of the eye and face. |
| 2. Contraction of the blood-vessels of the brain proper. | 2. Loss of consciousness, and accumulation of blood in the base of the encephalon. |
| 3. Extension of the first excitation, *partly* due to the accumulation of blood in the base of the encephalon. | 3. Tonic contraction of the laryngeal, the cervical, and the thoracic expiratory muscles. (*Laryngismus* and *trachelismus.*) |
| 4. Contraction of laryngeal and of thoracic expiratory muscles. | 4. Crying and stoppage of respiration. |
| 5. Farther extension of the first excitation of the nervous centre. | 5. Tonic contraction, extending to most of the muscles of the trunk and limbs. |
| 6. Loss of consciousness and tonic contraction in the trunk and limbs. | 6. Falling. |
| 7. Laryngismus, trachelismus, and the fixed state of the chest. | 7. Asphyxia, with obstacles to the return of venous blood from the head and the spinal cavity. |
| 8. Asphyxia, and the accumulation of black blood in the encephalon and in the spinal cord. | 8. *Clonic convulsions* everywhere; contractions of the bowels, the bladder, the uterus; erection; increase of many secretions; efforts at inspiration. |
| 9. Exhaustion of nervous power generally, and of the reflex faculty especially, except for respiration, which gradually becomes normal. | 9. Cessation of the convulsions; coma or heavy sleep, after which extreme fatigue and headache. |

Lecture XII. contains little else than discussions of physiological questions as to the relations of the medulla oblongata, the pons Varolii and certain parts of the spinal cord with the respiratory movements, convulsive rotatory acts, and other matters of more or less interest to the student of biology.

The appendix constitutes about one-fourth of the whole volume, and contains numerous interesting suggestions as to the treatment of injuries of the spine, burns, etc.

The only general fault to be found with the volume which we have thus briefly analyzed, is the unfortunate lack of methodical arrangement which characterizes it throughout, and which makes it a most fatiguing book to read, in despite of the interest which attaches to all that comes to us from the pen of its distinguished author. Its style it would be unfair to criticise, since M. Brown-Séquard can scarcely be held rigorously to the canons of a language which is not his birthright.

